# The Effect of Ozone Treatment on Metabolite Profile of Germinating Barley

**DOI:** 10.3390/foods11091211

**Published:** 2022-04-21

**Authors:** Xue Dong, Litao Sun, Manjree Agarwal, Garth Maker, Yitao Han, Xiangyang Yu, Yonglin Ren

**Affiliations:** 1 College of Science, Health, Engineering and Education, Murdoch University, 90 South Street, Murdoch, Perth 6150, Australia; 33523904@student.murdoch.edu.au (X.D.); litao.sun@murdoch.edu.au (L.S.); m.agarwal@murdoch.edu.au (M.A.); g.maker@murdoch.edu.au (G.M.); han.nelson@murdoch.edu.au (Y.H.); 2 Institute of Agricultural Resources and Environment, Jiangsu Academy of Agricultural Sciences, 50 Zhongling Street, Nanjing 210014, China

**Keywords:** ozone (O_3_), barley (*Hordeum vulgare* L.), germination, volatile organic compounds (VOCs), metabolites, head space solid-phase microextraction (HS-SPME), gas chromatography mass spectrometry (GC-MS)

## Abstract

Ozone is widely used to control pests in grain and impacts seed germination, a crucial stage in crop establishment which involves metabolic alterations. In this study, dormancy was overcome through after-ripening (AR) in dry barley seed storage of more than 4 weeks; alternatively, a 15-min ozone treatment could break the dormancy of barley immediately after harvest, with accelerated germination efficiency remaining around 96% until 4 weeks. Headspace solid-phase microextraction (HS-SPME) and liquid absorption coupled with gas chromatography mass spectrometry (GC-MS) were utilized for metabolite profiling of 2-, 4- and 7-day germinating seeds. Metabolic changes during barley germination are reflected by time-dependent characteristics. Alcohols, fatty acids, and ketones were major contributors to time-driven changes during germination. In addition, greater fatty acids were released at the early germination stage when subjected to ozone treatment.

## 1. Introduction

Barley (*Hordeum vulgare* L.) is a widely cultivated and highly adaptable crop predominantly used for stockfeed, food products, and the brewing industry through the malting process. In addition to a small amount of them being used as seed to establish the next season’s crop, harvested barley seeds are used for different purposes. The quality of barley significantly influences its end utilization since the germination rate of barley seed must be >95% for the malting process [1]. There are many reasons causing failure of germination. A failure of an intact viable seed to complete germination under favorable conditions is defined as seed dormancy [2]. Seed dormancy is a complex trait that is determined by genetic factors and substantial environmental conditions. Environmental factors such as moisture, temperatures, and light affect seed dormancy and regulate the timing of germination under natural conditions [3,4].

The majority of barley varieties have been bred with dormancy traits to prevent preharvest sprouting [5]; however, dormancy that persists after harvest delays germination resulting in yield and malting losses. There are different methods for eliminating seed dormancy, including chemical, mechanical, and biological scarification; stratification; phytohormone application; exposure to photoperiod and thermoperiod; and combinations of them [6,7,8]. Additionally, the potential for embryo dormancy can be overcome through after-ripening (AR) in dry seed storage [9], which has been demonstrated in many species, including *Arabidopsis thaliana*, wheat, and barley [10,11,12]. However, induction of secondary dormancy and deterioration can occur in seeds during dry storage [13].

Embryo dormancy is generally regulated by the content of phytohormones abscisic acid (ABA) and gibberellic acid (GA) [14,15]. It has been reported that ozone (O_3_) may trigger antioxidants that regulate hormone levels, particularly abscisic acid (ABA), to break tomato seed dormancy and enhance germination [16,17]. Our previous study demonstrated that both a greater variety and quantity of volatile organic compounds (VOCs) were released under oxidative stress and that acetic acid can regulate barley germination depending on the dosage [18]. However, metabolite changes during seed germination in response to ozone treatment remain elusive. Seed germination can be affected by intrinsic and extrinsic factors. The germination process is characterized by various metabolic processes resulting in distinct metabolic and time-dependent alterations. Seeds store food reserves such as proteins, carbohydrates, and lipids which are the source of energy, carbon, and nitrogen during germination and seedling establishment. Starch degradation into sugars and protein degradation into amino acids during seed germination have been extensively studied [19,20]. Lipids are a minor component of seeds, playing a vital role in the structure and function of cell membranes and acting as an energy store to allow metabolism to continue during abiotic stress. During germination, fatty acids are released through lipid mobilization and then degraded through the β-oxidation and glyoxylate cycles, and subsequently converted into sugars [21]. In addition, fatty acids are oxidized to hydroperoxide by lipoxygenase and then degraded to volatiles alkanes, aldehydes, ketones, and alcohols [22,23].

Numerous works focused on protein [24], transcripts [25], and primary metabolites [26,27] during barley germination, but the volatile metabolite profile of barley during germination is still unknown. In addition, metabolite changes in germinating seeds in response to ozone treatment remain elusive. This study aims to characterize VOCs and metabolites of germinating barley based on head space solid-phase microextraction (HS-SPME) and liquid absorption combined with gas chromatography-mass spectrometry (GC–MS). Furthermore, the impact of ozone on metabolite profiles in the germinating seeds will also be assessed.

## 2. Materials and Methods

### 2.1. Barley Seeds

Barley samples of varieties Scope CL and Flinders were harvested in 2020 from the site in Narrogin, Western Australia (32.94 S, 117.24 E), within 10.8% and 10.6% moisture content, respectively. The essential characteristics are shown in Table S1.

### 2.2. Ozone Generation

The continuous ozone gas was supplied by a commercial ozone generator (Model FH-CYJ1520A-20 g/h, maximum concentration = 700 ppm; Shanghai Fenghua Electronics Technology Co., Ltd., Shanghai, China). Ozonation of barley seeds was performed in a 2-L and 16.6-cm diameter Hysil semibatch reactor with a desiccation glass chamber. One kilogram of barley seeds was placed on a supporting iron wire gauze 5 cm above the reactor bottom in order to provide enough space for gas circulation. Gas was introduced from the ozone generator directly to the bottom of the reactor and passed through the seeds by a 1 cm diameter hose. An ozone monitor (Model SKY2000-03; Shenzhen Yuan Technology Co., Ltd., Shenzhen, China) was used to measure the ozone concentration to and from the reactor.

Seeds were introduced into the reactor and treated with different O_3_ treatment times at 700 ppm, T1 = 15 min, T2 = 120 min, while the air was passed for the control (C) barley seeds. After treatment, seeds were removed from the reactor, dried at room temperature, and stored in a closed glass container with 25 ± 1 °C, 11% RH for further study.

### 2.3. Germination Test

The germination tests were performed during different periods of rest time before the test: (1) immediately after treatment (T1, T2, and C); (2) 1 week after O_3_ treatment (1T1, 1T2, and 1C); (3) 2 weeks after O_3_ treatment (2T1, 2T2, and 2C); (4) 4 weeks after O_3_ treatment (4T1, 4T2, and 4C); and (5) 13 weeks after O_3_ treatment (13T1, 13T2, and 13C). These experimental conditions followed a complete factorial design with three independent factors: variety, treatment duration, and rest time before germination. The germination test was performed by the between-paper (BP) method of the International Seed Testing Association methods [28]. As shown in Figure S2, 424 (eight replicates of 53 seeds) of each treatment were randomly selected. A large filter paper was saturated with 60 mL distilled water and folded in half, creating a double thickness. Once the seeds were positioned correctly, the upper half of the filter paper was folded over the seed area [29]. All treatments were incubated for 7 days at 25 ± 1 °C in the dark. Visible radicle protrusion was used as the criterion for germination. The germination rate was detected 7 days after the germination test. Meanwhile, germinated seeds were collected for further metabolic profiling 2, 4, and 7 days after the start of the germination test.

### 2.4. Liquid absorption VOC Collection

Grain samples collected at 2, 4, and 7 d during germination were used for metabolic analysis and 0.35 g germinated seeds were placed in 2 mL Eppendorf microcentrifuge tubes. Then, 500 uL acetonitrile and 3 silicon beads were added to the vial and ground at 4000 rpm for 120 s. Next, 500 uL acetonitrile was added and vortexed at 4000 rpm for 60 s, then ground at 4000 rpm for a further 120 s. The solution was stood at room temperature for 1 h. After completion of the liquid extraction, 200 uL supernatant was transferred to a glass autosampler vial, with 1 μL of the liquid adsorbent being injected into the GC-MS by liquid autosampler.

### 2.5. HS-SPME VOC Collection

One gram samples of germinating barley seeds were transferred into 25 mL SPME vials with screw caps. A three-phase SPME fiber (50/30 μM) with a 2-cm combination coating of divinylbenzene/carboxen/polydimethylsiloxane (DVB/CAR/PDMS, 50/30 μM; Agilent Technologies, Santa Clara, CA, USA) was utilized for VOC absorption. After exposure to the headspace of the vial at 25 °C for 60 min, the fiber was removed from the vial and desorbed for 7 min onto the GC injection port operated in splitless mode. A vial containing moistened filter paper (deionized water) was used for control.

### 2.6. GC-MS Conditions

HS-SPME sample was measured by an Agilent 7890B GC system coupled with 5977B MSD. An Agilent HP-5MS capillary column (30 m length, 0.25 mm internal diameter, 0.25 μM film thickness, 5% phenyl and 95% dimethylpolysiloxane stationary phase) was employed for compound analysis. Ultra-high purity helium (Air Liquide, Perth, Australia) was the carrier gas at a constant flow rate of 1.0 mL/min. The temperature program started at 40 °C for 2 min, ramped at 5 °C/min to 180 °C, then at 20 °C/min to 260 °C and then held at 260 °C for 4 min. The detector was operated in electron impact (EI) mode at 70 eV and the mass range was from 40 to 400 atomic mass units (amu). The ion source temperature, quadruple temperatures, and transfer line temperature of the MSD were maintained at 230 °C, 150 °C, and 280 °C, respectively.

For liquid samples, the Agilent 7693A Automatic Liquid Sampler (ALS) GC system coupled with 5977E MSD was employed for compound analysis with the same column as HS-SPME sample analysis. The flow rate of carrier gas (helium, purity > 99.999%; Perth, Australia) was 1.0 mL/min. The oven temperature was 40 °C for 4 min, ramped at 5 °C/min to 180 °C, then 15 °C/min to 300 °C, and held for 4 min. The total analysis time was 44 min and the run was carried out with a solvent delay of 2 min. The electron ionization (EI) voltage was 70 eV and the spectra scan range was 40–600 atomic mass units (amu). The ion source temperature, quadruple temperatures, and transfer line temperature of the MSD were 230 °C, 150 °C, and 280 °C, respectively.

The individual peaks were categorized by comparison with NIST 05 NIST Mass Spectral. Compounds were identified by an experimentally obtained Kovats retention index (RI) of C7–40 alkane standards and mass spectra in the National Institutes of Standards and Technology Mass Spectrometry (NIST MS) library. 

### 2.7. Data Analysis

Data acquisition of GC-MS was processed using the Mass Hunter Acquisition software (vB.06.00; Agilent Technologies, Santa Clara, CA, USA). The partial least squares discriminant analysis (PLS-DA) with variable importance in projection (VIP) and cluster heatmap were performed by MetaboAnalyst 5.0 (https://www.metaboanalyst.ca/ (accessed on 21 January 2022)). One-way analysis of variance (ANOVA) was utilized to analyze the significant difference among treatments by SPSS (Version 25.0). Tukey’s post-hoc test (HSD) with *p*-value (α = 0.05) was employed. Figures were generated using Origin software (Version 2021b, OriginLab, Northampton, MA, USA). 

## 3. Results and Discussions

### 3.1. The Effect of Storage Time on Barley Germination

**Figure 1:**
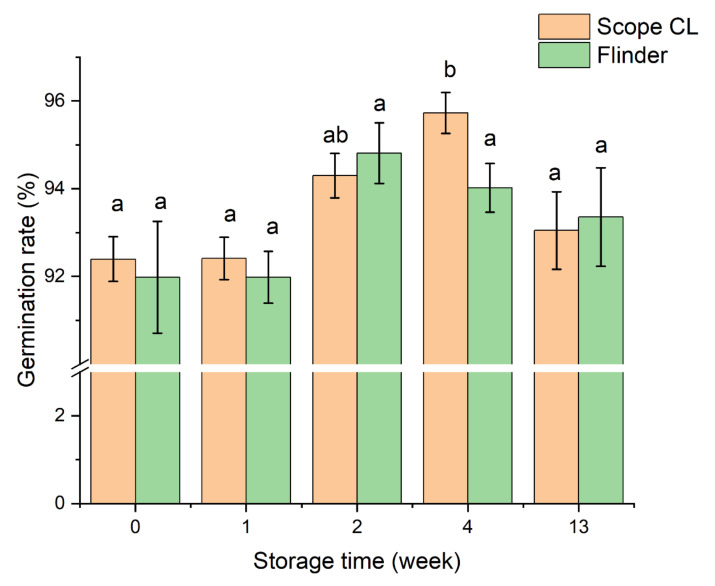
Effect of storage time on barley germination in Scope CL and Flinder varieties. 424 (eight replicates of 53 seeds) of each treatment were utilized. The data represent the average of eight replications, and the bars represent standard errors. Bars with different letters in each varieties are significantly different (Tukey’s HSD test, *p* < 0.05).

In this study, two different malting barley varieties were utilized for the germination test. To be used for malting, barley requires a germination rate greater than 95%. In this study, after the harvest, the germination rate of Scope CL and Flinder was 92.39% and 91.98%, respectively (Figure 1). Viable grain may not germinate under favorable conditions if the seed is dormant, which prevents preharvest sprouting in the field. However, dormancy that persists after harvest is undesirable because it prevents malting barley germination resulting in malting loss [30]. The germination data demonstrated that the general trend of two varieties’ germination ability gradually increased during storage. The germination rate of Scope CL significant increased from 92.39% to 95.72% after 4 weeks of storage, while that of Flinder increased from 91.98% to 94.81% after 2 weeks of storage, which is in accordance with previous research suggesting that during dry storage at room temperature, the germination percentage of seeds increases [31,32]. Primary dormancy is often lost during after-ripening in many species, including wheat and barley [12,33]. Peanut seeds increased ethylene production during after-ripening to relief dormancy [34]. Dormancy loss during after-ripening in sunflower seeds was correlated with a reduction in sensitivity to abscisic acid (ABA), an inhibitor of germination [35]. During after-ripening, wheat seeds became sensitive to treatment with gibberellin (GA), which promoted germination, and then became insensitive to ABA [15]. Although much progress has been made in understanding after-ripening, the specific mechanisms of dormancy alleviation by after-ripening remain poorly understood.

### 3.2. Influence of Ozone in Barley Seed Dormancy Alleviation

**Figure 2:**
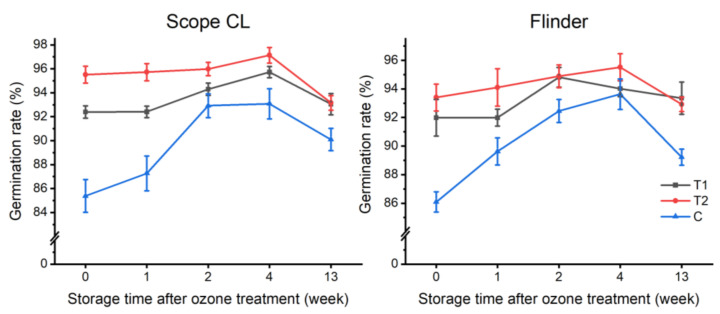
The effect of ozone treatments at different time on seed dormancy alleviation based on three independent factors, i.e., variety (Scope CL and Flinder), ozone treatment duration (T1 = 15 min, T2 = 120 min of 700 ppm ozone treatment, C = control), and storage time after ozone treatment (immediately, 1, 2, 4, and 13 weeks of storage). The data represent the average of eight replications, and the bars represent standard errors.

The effect of O_3_ on seed dormancy alleviation is presented in Figure 2. The data demonstrated that a 15-min O_3_ treatment at 700 ppm promoted the germination ability of Scope CL and Flinder to 95.52% and 93.40%, respectively (the germination test was performed immediately after treatment). Samples treated with O_3_ for 15 min remained at the accelerated germination efficiency during storage up to 4 weeks. In terms of Scope CL, the germination rate of 1T1, 2T1, and 4T1 was enhanced to 95.52%, 95.99%, and 96.46%, respectively, but 13T1 did not show sustained germination acceleration efficiency. Flinder showed the same tendency. The germination rate of 1T1, 2T1, and 4T1 was enhanced to 94.10%, 94.88%, and 95.52%, respectively; however, 13T1 decreased to 92.92%.

Some studies have indicated that O_3_ can accelerate the germination of tomato seeds because reactive oxygen species (ROS) produced by O_3_ promote cell signaling (Sudhakar et al., 2011). Ozone could also induce gene expression involving GA synthesis (*GA20ox1*) and ABA catabolism (*ABA8′OH*) [36]. Some studies suggest that O_3_ promotes VOC release, such as acetic acid acetaldehyde, ethanol, and ethyl acetate, which were highly correlated with seed germination [37]. Seeds treated for 120 min were found to be injured, with the seeds recovering from injury as time progressed. Initially, the germination rate of Scope CL and Flinder was 85.38% and 86.09%, respectively, when subjected to a 120-min ozone treatment. However, it gradually increased to 93.08% and 93.63%, respectively, after 4 weeks of storage, indicating that, as time progressed, O_3_ treatment loses its effect.

### 3.3. The Changes in Metabolite Profiles during Germination and Their Response to Ozone Treatment

**Figure 3:**
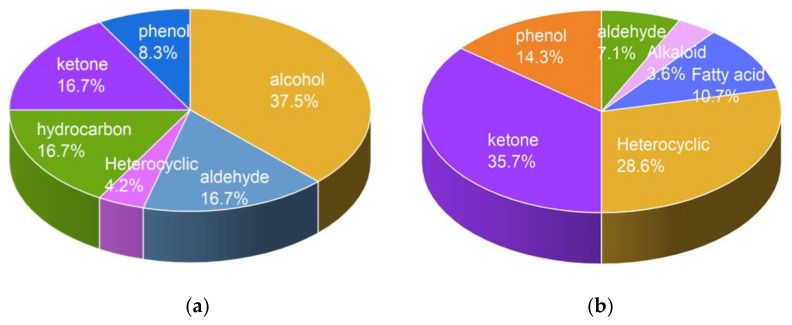
Chemical patterns of germinating barley seeds (Scope CL) based on (**a**) HS-SPME and (**b**) liquid absorption coupled with GC-MS. The data analyzed by three replications. Each area represents the percentage of the number compounds in the specific group.

In this study, the dormancy of both Scope CL and Flinder was reversed with 15-min ozone treatments and the germination rates of Scope CL in 1 week after a 15-min ozone treatment showed the most significant increase compared with control. Therefore, the Scope CL sample after one week of storage was utilized for VOC profiling. For GC-MS data, peaks were integrated into the total ion chromatogram (TIC). Any peak with more than 5000 total peak area was considered as detected and subsequently integrated. A total of 24 and 27 volatile compounds in barley seeds were identified by SPME and liquid adsorption methods based on retention time and mass spectral data from the NIST Mass Spectral Library. Metabolite profiling data were normalized by generalized logarithm transformation (Table 1 and Table 2). As shown in Figure 3A, identified compounds based on HS-SPME can be classified into six chemical groups. Alcohols, hydrocarbons, and aldehydes were the dominant groups, representing about 28.6%, 23.8%, and 19% of the total number of VOCs, respectively, followed by ketones, phenols, and heterocyclics, accounting for 14.3%, 9.5%, and 4.8%, respectively. However, compounds obtained by liquid absorption showed different chemical patterns. Ketones, heterocyclics, and phenols were the main groups, occupying 35.7%, 28.6%, and 14.3%, respectively, followed by fatty acids, aldehydes, and alkaloids, representing about 10.7%, 7.1%, and 3.6%, respectively (Figure 3B). In this study, alcohols and hydrocarbons were obtained only by HS-SPME. Volatile alcohols regarded as potential aroma-active compounds generated from common lipid oxidation are easily extracted by HS-SPME due to their high volatility. Fatty acids were only extracted by liquid absorption, which meant that the HS-SPME method with a 25 °C extraction temperature might not allow the trapping of sufficient fatty acids. Hordenine was also detected only by the liquid absorption method. As an active compound during barley germination, hordenine has numerous health benefits, including inhibiting melanin content, promoting weight loss, and potential anticancer activity [38,39]. Compounds obtained from both methods were combined for further analysis.

**Figure 4:**
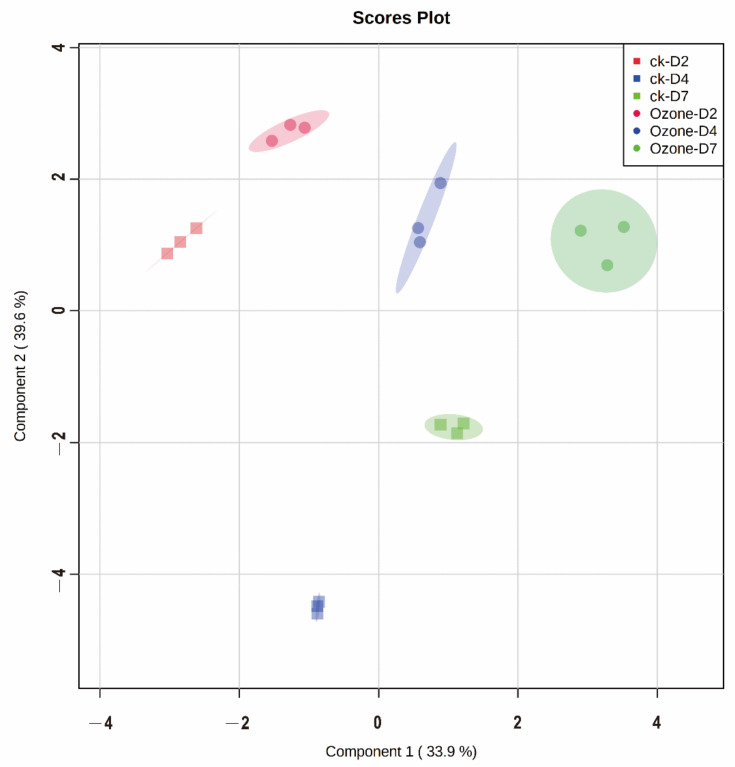
Partial least squares discriminant analysis (PLS-DA) scores plot for metabolite profiles of germinating barley with ozone treatment (control and 15 min of ozone treatment).

Since oxidative conditions resulted in high variability in barley VOC profiles based on our previous research, partial least squares discriminant analysis (PLS-DA) was carried out to evaluate variation among germinating barley with ozone treatment. In the original data, metabolites with more than 50% missing values were removed from subsequent analysis; the expression of metabolites was normalized by log-transformation and mean-centered scaling. Compounds presented variation in different germinating times, as shown by the complete separation among metabolite samples in the PLS-DA, indicating that considerable metabolic changes happened during germination. The first two principal components (PCs) explained 73.5% of the total variance with 33.9% of PC1 and 39.6% of PC2, and the scores plot emphasized the separation between barley samples among different germinating days (2, 4, and 7 days after imbibition) (Figure 4.)

Barley samples at different germination stages were separated along PC1, and ozone-treated and control samples were separated along PC2. The difference in metabolite profiles between ozone-treated and control samples started at 2 days and reached the highest 4 days during germination, suggesting ozone played a critical role in metabolite changes during germination, especially at 4 days. Clear separation among the treatments (CK and ozone treatment) was also detected at 7 days during germination, indicating that ozone treatments substantially affected germination patterns.

**Figure 5:**
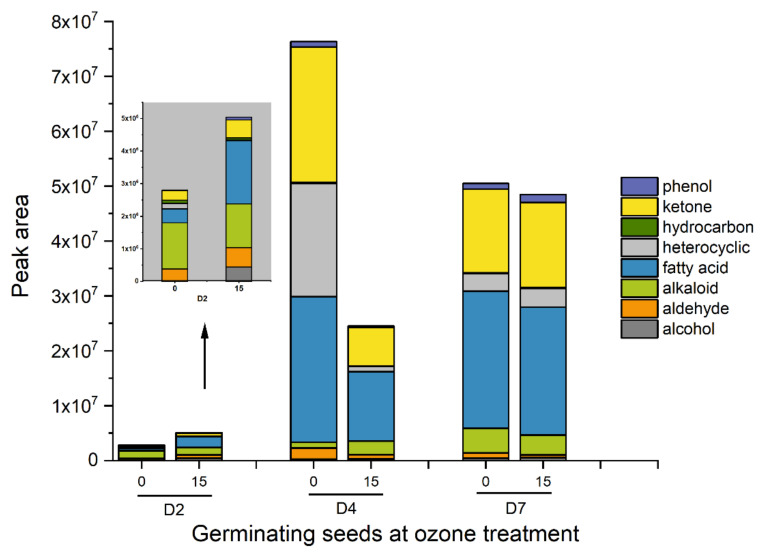
Volatile compound composition of germinating seeds (2, 4, and 7 days during germination) at ozone (0 and 15 min) treatment. The compounds of three replications were analyzed using solid-phase microextraction (SPME) and liquid absorption method.

Chemical groups in barley seeds showed various patterns during the germination process. Fatty acids, alcohols, and ketones were major contributors to time-driven changes during germination (Figure 5). The data demonstrated that more fatty acids were released from days 2 to 7 in both ozone-treated and control samples. Seeds contain lipids for energy storage, and the mobilization of storage lipid during seed germination begins with the hydrolysis of the glyceride into free fatty acids and glycerol [40]. Here, the ozone-treated sample produced a greater quantity of fatty acids 2 days after imbibition, but fewer fatty acids 4 days after imbibition compared with the control. As an oxidizing agent, ozone could promote lipid oxidation to release more fatty acids and alcohols at an early stage. Subsequently, fatty acid could be broken down into acetyl coenzyme A (acetyl-CoA) through β-oxidation and enter the citric acid cycle (TCA cycle) to supply carbon skeletons and energy for germination [41].

### 3.4. Dynamic Changes of Metabolites in Barley during Germination

**Figure 6:**
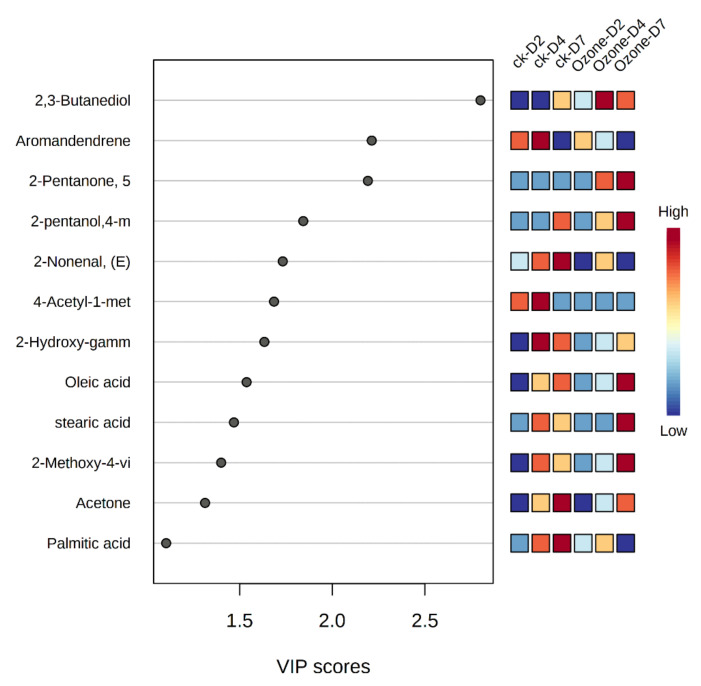
Variable importance in the projection (VIP) scores for germinating barley at zone treatment. VOCs was shown above with variable importance in projection (VIP) > 1.2. Red means a higher abundance of metabolites and blue means a lower abundance.

Metabolic responses during germination are presented in a heatmap (Figure S1). Variable importance in projection (VIP) reflects the importance of variables in PLS-DA classification. Integrating the results of the statistical analyses ANOVA and VIP score, 12 VOCs with FDR < 0.05 and VIP > 1.2 were selected, including two alcohols, three acetic acids, four ketones, one aldehyde, one phenol, and one hydrocarbon (Figure 6). 2,3-butanediol only existed in control samples at 7 days of germination; however, it existed in ozone-treated samples at 2, 4, and 7 days of germination and increased during the germination process (Table 1). 2,3-butanediol is a crucial volatile component detected in barley, which has been reported to enhance seed germination and seedling growth [42]. Ozone treatment induced more 2,3-butanediol (Figure 6), which could facilitate barley germination ability. In addition, oleic acid and stearic acid are the main components of germinating barley, which increased in both ozone-treated and control samples during germination. The increase in fatty acids may have been due to glyceride hydrolysis. The same variation was also observed in peanut and foxtail millet germination [43,44]. Palmitic acid increased and reached the maximum at 7 days germination in control samples, but 4 days germination in ozone-treated samples. Palmitic acid is a long chain fatty acid, playing a vital role in regulating energy metabolism. Ozone induced greater palmitic acid levels at an earlier stage compared to control, which might be attributed to enhanced germination ability by supplying more energy. Acetone was not detected at day 2 germination, appeared at day 4, and then increased at day 7 in barley germination, in accordance with trends observed in lima beans [45]. In addition, acetone production in seeds was closely correlated with lipid metabolism during germination [45]. As an aldehyde in barley seeds contributing to rancidity in beer [46], (E)-2-nonenal is derived from enzymatic or nonenzymatic oxidation of lipids and fatty acids [47]. Here, (E)-2-nonenal increased during germination; however, the ozone-treated sample showed a reduction of (E)-2-nonenal at day 7 germination, suggesting ozone could be applied for eliminating off-flavors of grains. 2-methoxy-4-vinylphenol gradually increased during germination. It is an aromatic substance responsible for the natural aroma [48], which could induce exogenous dormancy in wheat seeds [49]. Aromadendrene is not sufficiently studied in the literature, so will not be further discussed.

**Figure 7:**
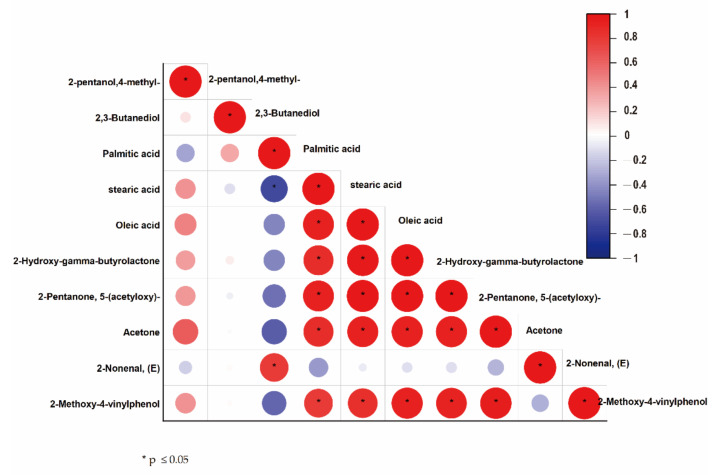
Correlation analysis of differential compounds in ozone-treated germinating barley. Correlation coefficient (positive or negative) between a pair of metabolites is represented by color based on Pearson correlation analysis. Red means positive correlation while blue represents negative. Asterisk means *p* value ≤ 0.05.

Correlation analysis was used to identify metabolites that were functionally related or coregulated in germinating barley seeds (Figure 7). Ten differential metabolites obtained above were included, and 100 metabolite pairs were analyzed. The results indicated oleic acid and stearic acid were highly positively correlated with each other. In addition, oleic acid and stearic acid were positively correlated to ketones including 5-(acetyloxy)-2-pentanone, 2-hydroxy-gamma-butyrolactone, and acetone, while palmitic acid showed significant negative correlations with the above ketones and positive correlation with (E)-2-nonenal. 5-(acetyloxy)-2-pentanone, 2-hydroxy-gamma-butyrolactone, and acetone were also highly positively correlated with each other. This result suggests that ozone induced an increase in fatty acids and ketones during germination, while ozone treatment appeared to induce conversion of metabolic products from palmitic acid to ketones.

## 4. Conclusions

In this study, dormancy in two varieties of malting barley persisted after harvest and was lost during after-ripening up to 4 weeks; however, a 15-min ozone treatment could break dormancy of barley immediately and it remained at the accelerated germination efficiency ~96% until 4 weeks. HS-SPME and liquid absorption coupled with GC–MS were utilized for metabolite profiling. Ketones, alcohols, and fatty acids contributed to a variation in different germinating times of barley. Ozone also played an important role in metabolite changes during germination, especially at day 4 of germination. In addition, ozone induced an increase of fatty acid levels to enhance germination by supplying carbon skeletons and energy for germination via TCA cycle.

## Figures and Tables

**Table 1:** Analysis of volatile compounds of germinating barley seeds (Scope CL) under different ozone treatment times by HS-SPME-GC-MS.

Chemical Groups	Compounds	RT *^a^*	RI (lib) *^b^*	RI (cal) *^c^*	Peak Area (log _10_)					
					Control (Ozone (0 min))			Ozone (15 min)		
					D2	D4	D7	D2	D4	D7
Alcohol	2-Propanol, 1-methoxy-	4.11	661		n.d. *^d^*	n.d.	n.d.	5.44 ± 0.40 ^*e*^	n.d.	n.d.
	1-Butanol, 3-methyl-	5.45	736	732	n.d.	4.32 ± 0.21	4.74 ± 0.04	n.d.	n.d.	4.53 ± 0.06
	1-Butanol, 2-methyl-	5.54	739	736	n.d.	4.18 ± 0.24	4.19 ± 0.03	n.d.	4.01 ± 0.19	4.37 ± 0.07
	2-Pentanol,4-methyl-	6.67	758	778	n.d.	n.d.	4.67 ± 0.31	n.d.	4.63 ± 0.24	4.90 ± 0.86
	2,3-Butanediol	7.01	788	791	n.d.	n.d.	4.80 ± 0.09	4.55 ± 0.44	5.12 ± 0.36	4.94 ± 0.09
	3-Hexen-1-ol, (Z)	9.02	839	856	n.d.	4.93 ± 0.39	5.21 ± 0.20	n.d.	n.d.	4.91 ± 0.01
	1-Hexanol	9.42	868	868	n.d.	n.d.	4.51 ± 0.04	n.d.	n.d.	n.d.
	1-Hexanol, 2-ethyl-	14.67	1030	1029	n.d.	4.68 ± 0.15	n.d.	n.d.	n.d.	4.36 ± 0.17
	2-Nonen-1-ol, (E)-	19.09	1169	1171	4.23 ± 0.14	4.58 ± 0.34	4.80 ± 0.14	4.63 ± 0.28	4.82 ± 0.06	n.d.
Aldehyde	Acetaldehyde	2.75			n.d.	5.20 ± 0.09	n.d.	n.d.	5.07 ± 0.26	n.d.
	Hexanal	7.26	801	801	4.24 ± 0.16	4.96 ± 0.06	4.60 ± 0.22	4.65 ± 0.24	4.37 ± 0.04	4.61 ± 0.23
	Octanal	13.85	1003	1003	n.d.	n.d.	4.53 ± 0.18	n.d.	n.d.	n.d.
	2-Nonenal, (E)-	18.82	1162	1162	3.70 ± 0.14	4.90 ± 0.15	5.46 ± 0.17	n.d.	4.58 ± 0.48	n.d.
Heterocyclic	Furan, 2-pentyl-	13.48	993	995	3.90 ± 0.16	4.53 ± 0.20	4.28 ± 0.08	4.09 ± 0.25	3.81 ± 0.21	4.34 ± 0.10
Hydrocarbon	7-Tetradecene	24.33	1369	1356	n.d.	4.49 ± 0.14	4.59 ± 0.21	n.d.	3.71 ± 0.05	4.55 ± 0.09
	(-)-Aristolene	26.39	1453	1435	4.30 ± 0.14	4.62 ± 0.27	4.35 ± 0.08	4.35 ± 0.09	4.05 ± 0.07	4.66 ± 0.02
	Aromadendrene	26.58	1461	1443	4.41 ± 0.38	4.84 ± 0.30	n.d.	4.13 ± 0.19	3.65 ± 0.10	n.d.
	β-Guaiene	26.87	1490	1454	4.70 ± 0.12	4.58 ± 0.04	4.28 ± 0.07	4.53 ± 0.12	4.26 ± 0.14	4.57 ± 0.11
Ketone	Acetone	2.02			n.d.	5.62 ± 0.07	6.31 ± 0.12	n.d.	5.48 ± 0.10	6.30 ± 0.13
	Acetoin	4.91	713	712	n.d.	5.82 ± 0.24	6.12 ± 0.14	n.d.	5.99 ± 0.39	5.72 ± 0.09
	5-Hepten-2-one, 6-methyl-	13.34	986	991	n.d.	4.42 ± 0.25	n.d.	4.34 ± 0.07	n.d.	n.d.
	4-Acetyl-1-methylcyclohexene	17.43	1110	1116	4.36 ± 0.14	4.76 ± 0.06	n.d.	n.d.	n.d.	n.d.
Phenol	Phenol	13.07	981	982	4.08 ± 0.28	n.d.	n.d.	4.39 ± 0.19	n.d.	n.d.
	2,4-Di-tert-butylphenol	28.39	1519	1515	n.d.	4.51 ± 0.26	n.d.	n.d.	n.d.	n.d.

*^a^* Retention time. *^b^* Retention index by searching NIST library. *^c^* Retention index calculated by C_7_–C_40_ alkanes external standards. *^d^* Not detected. *^e^* Mean of three replications ± standard error.

**Table 2:** Analysis of volatile compounds of germinating barley seeds (Scope CL) under different ozone treatment times by liquid absorption.

Chemical Groups	Compounds	RT *^a^*	RI (lib) *^b^*	RI (cal) *^c^*	Control (Ozone (0 min))			Ozone (15 min)		
					D2	D4	D7	D2	D4	D7
Aldehyde	Benzeneacetaldehyde	17.06	1045	1047	n.d. *^d^*	6.09 ± 0.04 ^*e*^	5.47 ± 0.02	5.45 ± 0.29	5.57 ± 0.17	5.30 ± 0.26
	2-Decenal, (Z)-	23.70	1252	1263	5.25 ± 0.06	5.65 ± 0.10	5.43 ± 0.01	5.11 ± 0.01	5.18 ± 0.07	5.16 ± 0.03
	2-Undecenal	26.52	1367	1365	5.20 ± 0.05	n.d.	n.d.	4.95 ± 0.04	4.74 ± 0.01	5.01 ± 0.13
Alkaloid	Hordenine	29.21	1495	1479	6.15 ± 0.01	6.02 ± 0.03	6.63 ± 0.16	6.13 ± 0.06	6.38 ± 0.12	6.55 ± 0.08
Fatty acid	Acetic acid	5.18	610		4.94 ± 0.09	7.19 ± 0.03	7.04 ± 0.05	n.d.	6.74 ± 0.05	7.01 ± 0.05
	Palmitic acid	36.87	1968	1962	5.43 ± 0.01	6.85 ± 0.02	6.91 ± 0.06	6.12 ± 0.21	6.62 ± 0.08	n.d.
	Oleic acid	38.23	2133	2141	5.20 ± 0.07	6.55 ± 0.08	6.73 ± 0.01	5.71 ± 0.12	6.44 ± 0.13	6.88 ± 0.01
	stearic acid	38.37	2172	2161	4.36 ± 0.17	5.55 ± 0.02	5.53 ± 0.05	n.d.	n.d.	6.73 ± 0.09
Heterocyclic	2-Furanmethanol	10.88	860	869	n.d.	6.22 ± 0.08	5.67 ± 0.18	n.d.	5.66 ± 0.13	5.52 ± 0.44
	2-furancarboxaldehyde, 5-methyl	14.30	964	965	n.d.	5.82 ± 0.12	5.33 ± 0.04	n.d.	n.d.	5.44 ± 0.12
	2,4-Dihydroxy-2,5-dimethyl-3(2H)-furan-3-one	14.98	989	984	4.61 ± 0.23	6.29 ± 0.13	5.76 ± 0.19	n.d.	5.18 ± 0.25	5.73 ± 0.40
	Furaneol	17.61	1070	1063	n.d.	6.37 ± 0.01	5.90 ± 0.02	n.d.	5.48 ± 0.05	5.79 ± 0.23
	Maltol	19.30	1110	1116	5.19 ± 0.02	6.24 ± 0.03	5.12 ± 0.03	n.d.	n.d.	5.50 ± 0.06
	2(3H)-Furanone, dihydro-4-hydroxy-	20.68	1185	1161	4.34 ± 0.10	6.49 ± 0.18	5.62 ± 0.07	n.d.	n.d.	5.68 ± 0.19
	5-Hydroxymethylfurfural	22.72	1232	1229	4.55 ± 0.09	6.94 ± 0.08	5.76 ± 0.17	n.d.	n.d.	5.61 ± 0.19
Ketone	2-Propanone,1-hydroxy	5.87	666		4.66 ± 0.02	6.76 ± 0.01	6.74 ± 0.09	n.d.	6.51 ± 0.06	6.71 ± 0.02
	4-Cyclopentene-1,3-dione	11.51	883	886	n.d.	6.16 ± 0.07	5.67 ± 0.01	n.d.	5.49 ± 0.15	5.68 ± 0.20
	2-Cylopenten-1-one, 2-hydroxy-	13.13	926	932	5.06 ± 0.15	6.44 ± 0.10	5.99 ± 0.05	5.38 ± 0.17	5.72 ± 0.11	5.93 ± 0.21
	2-Hydroxy-gamma-butyrolactone	15.36	1011	995	6.30 ± 0.13	6.42 ± 0.01	6.18 ± 0.02	5.16 ± 0.35	5.78 ± 0.05	6.16 ± 0.07
	1,2-Cyclopentanedione,3-methyl-	16.54	1028	1031	5.72 ± 0.09	n.d.	n.d.	n.d.	n.d.	5.25 ± 0.14
	2-Pentanone, 5-(acetyloxy)-	17.32	1053	1055	n.d.	n.d.	n.d.	n.d.	4.94 ± 0.19	5.53 ± 0.05
	4H-Pyran-4-one, 2,3-dihydro-3,5-dihydroxy-6-methyl-	20.27	1150	1147	n.d.	7.00 ± 0.07	6.49 ± 0.13	n.d.	5.79 ± 0.06	6.58 ± 0.20
	4H-Pyran-4-one, 3,5-dihydroxy-2-methyl-	21.53	1196	1188	n.d.	5.73 ± 0.10	n.d.	n.d.	n.d.	5.36 ± 0.08
	2-Heptadecanone	36.36	1904	1903	5.18 ± 0.01	5.18 ± 0.01	n.d.	4.97 ± 0.09	4.70 ± 0.02	n.d.
Phenol	4-Vinylphenol	22.38	1223	1217	n.d.	5.54 ± 0.01	5.27 ± 0.12	n.d.	n.d.	5.32 ± 0.20
	2-Methoxy-4-vinylphenol	25.25	1316	1318	n.d.	5.44 ± 0.07	5.39 ± 0.18	4.76 ± 0.13	5.01 ± 0.08	5.58 ± 0.17
	Phenol, 4-ethenyl-2,6-dimethoxy-	31.68	1567	1584	n.d.	5.52 ± 0.05	5.78 ± 0.13	n.d.	5.23 ± 0.04	5.92 ± 0.02

*^a^* Retention time. *^b^* Retention index by searching NIST library. *^c^* Retention index calculated by C_7_–C_40_ alkanes external standards. *^d^* Not detected. *^e^* Mean of three replications ± standard error.

## Data Availability

The data presented in this study are available within the article.

## Supplementary Materials

The following supporting information can be downloaded at: https://www.mdpi.com/article/10.3390/foods11091211/s1, Figure S1: Heatmap of metabolites at different stages of germination. Red means a higher abundance of metabolites and blue means a lower abundance; Figure S2: Diagram of germination test and sample collection; Table S1: Basic information of the two varieties of barley seeds used in this study.
