# Supplementary material for: The Effect of Ozone Treatment on Metabolite Profile of Germinating Barley
Source: Foods. 2022 Apr 21;11(9):1211. doi: 10.3390/foods11091211 (PMC9104593; doi:10.3390/foods11091211)
Supplement: Supplementary file 1 [file foods-11-01211-s001.zip › foods-1662682-supplementary.pdf]

Supplementary Materials

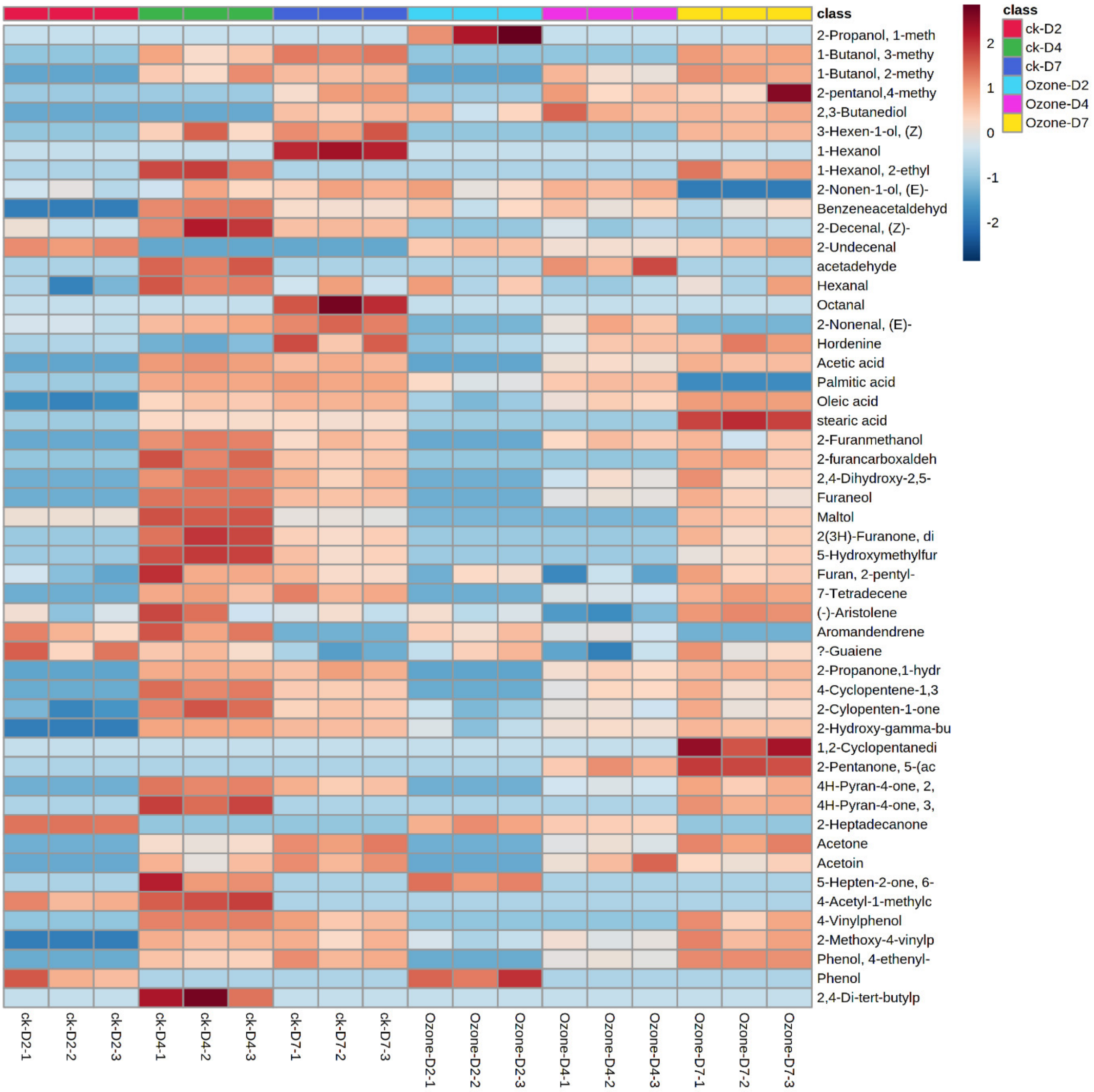

Figure S1. Heatmap of metabolites at different stages of germination. Red means a higher abundance of metabolites, blue means a lower abundance.

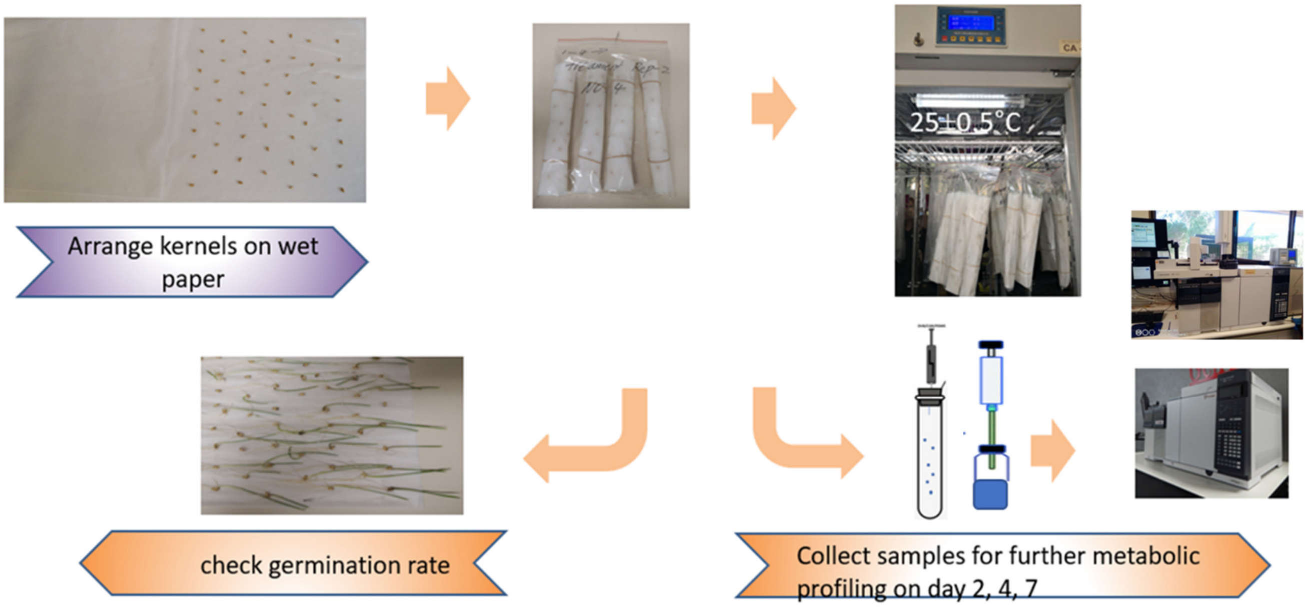

Figure S2. Diagram of germination test and sample collection

Table S1. Basic information of two variety of barley seeds

Properties	Scope CL	Flinders
Harvest time	2020	2020
Moisture content (%)	10.80	10.60
Thousand grain weight (g)	50.00	40.90
Germination rate	92.90%	91.90%
